# Accelerated Ovarian Failure as a Unique Model to Study Peri-Menopause Influence on Alzheimer’s Disease

**DOI:** 10.3389/fnagi.2019.00242

**Published:** 2019-09-06

**Authors:** Roberta Marongiu

**Affiliations:** Laboratory of Molecular Neurosurgery, Weill Cornell Medicine, Department of Neurosurgery, Cornell University, New York, NY, United States

**Keywords:** Alzheimer’s disease, menopause, peri-menopause, accelerated ovarian failure, ovariectomy (OVX), reproductive senescence

## Abstract

Despite decades of extensive research efforts, efficacious therapies for Alzheimer’s disease (AD) are lacking. The multi-factorial nature of AD neuropathology and symptomatology has taught us that a single therapeutic approach will most likely not fit all. Women constitute ~70% of the affected AD population, and pathology and rate of symptoms progression are 2–3 times higher in women than men. Epidemiological data suggest that menopausal estrogen loss may be causative of the more severe symptoms observed in AD women, however, results from clinical trials employing estrogen replacement therapy are inconsistent. AD pathological hallmarks—amyloid β (Aβ), neurofibrillary tangles (NFTs), and chronic gliosis—are laid down during a 20-year prodromal period before clinical symptoms appear, which coincides with the menopause transition (peri-menopause) in women (~45–54-years-old). Peri-menopause is marked by widely fluctuating estrogen levels resulting in periods of irregular hormone-receptor interactions. Recent studies showed that peri-menopausal women have increased indicators of AD phenotype (brain Aβ deposition and hypometabolism), and peri-menopausal women who used hormone replacement therapy (HRT) had a reduced AD risk. This suggests that neuroendocrine changes during peri-menopause may be a trigger that increases risk of AD in women. Studies on sex differences have been performed in several AD rodent models over the years. However, it has been challenging to study the menopause influence on AD due to lack of optimal models that mimic the human process. Recently, the rodent model of accelerated ovarian failure (AOF) was developed, which uniquely recapitulates human menopause, including a transitional peri-AOF period with irregular estrogen fluctuations and a post-AOF stage with low estrogen levels. This model has proven useful in hypertension and cognition studies with wild type animals. This review article will highlight the molecular mechanisms by which peri-menopause may influence the female brain vulnerability to AD and AD risk factors, such as hypertension and apolipoprotein E (APOE) genotype. Studies on these biological mechanisms together with the use of the AOF model have the potential to shed light on key molecular pathways underlying AD pathogenesis for the development of precision medicine approaches that take sex and hormonal status into account.

## Introduction

Alzheimer’s disease (AD) is an irreversible, progressive neurodegenerative disorder and a leading cause of mortality (Alzheimer’s Association, 2016; Scheltens et al., 2016). AD accounts for approximately 60%–80% of dementia cases in older adults with an average age at onset of around 65 years of age (Alzheimer’s Association, 2016). Symptoms are characterized by memory loss and impairment in other non-memory aspects of cognitive function such as word-finding, vision/spatial issues, and impaired reasoning or judgment. This loss of cognitive functioning along with loss of behavioral abilities are extremely debilitating for AD patients and dramatically interfere with their daily life (Scheltens et al., 2016).

Neuropathologically, AD is characterized by progressive deposition of parenchymal senile plaques comprised of amyloid-β (Aβ) protein, intracellular neurofibrillary tangles (NFTs) of abnormal phosphorylated tau, and chronic gliosis primarily in the hippocampus and neocortex (Price et al., 1991; Braak and Braak, 1997; Montine et al., 2012; Au et al., 2016; Scheltens et al., 2016; Lane et al., 2018), brain areas critical for learning and memory. This leads to synaptic damage, cognitive impairment, and neurodegeneration (Canter et al., 2016; Scheltens et al., 2016).

Cause(s) of AD are still unknown but several gene mutations, as well as other genetic and modifiable risk factors, have been identified. Age is the major risk factor for sporadic AD. As the number of aging Americans rapidly increases, so does the incidence of AD. An estimated 47 million people are currently living with AD worldwide, with 5.8 million people only in the US. This number is projected to triple by 2,050 to over 16 million (Alzheimer’s Association, 2016; Deb et al., 2017; Nebel et al., 2018). The ongoing demographic changes as well as the lack of treatments and preventive strategies will contribute not only to the rising of AD prevalence, but also to the increasing medical costs associated with the disease, which will exceed $1 trillion (Alzheimer’s Association, 2016; Deb et al., 2017; Nebel et al., 2018).

Although remarkable research advancements were made in the past decade, the complexity of the cellular mechanisms underlying AD pathogenesis and neuropathology as well as the heterogeneity of symptoms among AD patients caused many clinical therapeutic trials to fail. Understanding the multi-factorial nature of AD pathogenesis and pathology is a crucial step to develop efficacious prevention and treatment strategies, and to optimize care and reduce high costs associated with the disease.

Women constitute nearly 70% of the affected AD population (Fisher et al., 2018), ~3.5 million Americans aged 65 or older. AD prevalence is 2–3 times higher in post-menopausal women than men, even after controlling for lifespan (Alzheimer’s Association, 2016; Laws et al., 2016; Scheltens et al., 2016; Fisher et al., 2018). Furthermore, women present with a faster pathology progression and greater memory impairment than men (Alzheimer’s Association, 2016; Laws et al., 2016; Fisher et al., 2018). Despite the established vulnerability, the biological mechanisms underlying the increased risk of AD in women are largely unknown. Clinical data suggest that loss of estrogen at menopause may be a main factor influencing susceptibility to AD, but results from clinical trials employing estrogen replacement therapy in post-menopausal women are inconsistent (Alzheimer’s Association, 2016; Fisher et al., 2018). Nevertheless, results from recent clinical studies have suggested that hormone replacement therapy (HRT) initiated during peri-menopause may lower the risk of dementia and have cognitive benefits (Henderson et al., 2005; Whitmer et al., 2011; Shao et al., 2012).

Pre-clinical rodent models have proven to be important for understanding the wide range of estrogen effects that occur during human menopause. Rodent models have been constructed to replicate different elements of human menopause. These include natural aging, ovariectomy (OVX) and hormone replacement, and genetic models (Marques-Lopes et al., 2018). Although the current rodent models of menopause have and will continue to inform our understanding of the role of both estrogen and HRT in menopause, they fail to adequately recapitulate the human menopause process. The intact aging model fails to achieve very low estrogen levels, and the OVX model lacks a stage mimicking peri-menopause. Among the pre-clinical models, the innovative accelerated ovarian failure (AOF) using the chemical 4-vinylcyclohexene diepoxide (VCD), uniquely recapitulates hormonal changes that occur during human menopause, including estrous acyclicity and fluctuation (as in human peri-menopause), followed by undetectable, estrogen levels (as in human post-menopause). These model also allow for the dissociation of the effects of hormone levels from the effects of aging in young animals (reviewed in Van Kempen et al., 2011).

Over the last 20 years, striking advancements have been made in our understanding of AD sex dimorphism. The overarching goal of this review article is to highlight recent advancements in understanding the molecular mechanisms by which menopause may influence the female brain vulnerability to AD in view of the rodent models of menopause available. Studies on these biological mechanisms in combination with the use of the AOF model have the potential to shed light on key signaling pathways with the potential to improve diagnosis, prevention or stabilization of risk factors, and clinical outcomes.

## The Human Menopause Process

Menopause, or cessation of menstrual cycling, is a uniquely human process and marks the beginning of women’s reproductive senescence (Walker and Herndon, 2008; Alberts et al., 2013). The majority of women enter menopause *via* a gradual and irreversible process (peri-menopause) of reduction in ovarian function and decline in estrogen levels followed by a decrease in estrogen receptor (ER) expression over several years. Duration of peri-menopause is ~5 years, between ages of 45 and 54, which is followed by amenorrhea and then post-menopause (Practice Committee of the American Society for Reproductive Medicine, 2008; Butler and Santoro, 2011; Harlow et al., 2012). Peri-menopause is marked by irregular estrous cycles and fluctuations in ovarian hormones, but not loss of estrogen levels which are typical of post-menopause (Harsh et al., 2007). This period of irregular hormone-receptor imbalance contributes to the physiological and psychological symptoms associated with menopause (Morrison et al., 2006; Practice Committee of the American Society for Reproductive Medicine, 2008). Although clinically menopause is primarily defined as reproductive senescence, biological changes occur that significantly alter brain function which is reflected by a range of neurological symptoms including depression, insomnia, hypertension, and cognitive dysfunction (Morrison et al., 2006; Practice Committee of the American Society for Reproductive Medicine, 2008).

With current age of menopause transition around 50 years and life expectancy getting close to 80 years, it is estimated that by 2,060 20%–30% of women in the US will be in post-menopause, meaning that a women will spend approximately 1/3 of her life in post-menopause (U.S. Census Bureau, 2017). Mounting evidences indicate that menopause constitutes a risk factor for developing AD and newly published work suggests that peri-menopause, as a neurological transition state, may be the key time for the female brain susceptibility to the disease (Brinton et al., 2015).

## Menopause Influence on Clinical Presentation of Alzheimer’s Disease

There is epidemiological, clinical, and biological evidence that sex dimorphism influences the onset, progression, and clinical manifestation of AD (Mazure and Swendsen, 2016; Fisher et al., 2018; Nebel et al., 2018). It is recognized that aging is the major risk factor for AD (Mielke et al., 2014; Alzheimer’s Association, 2016). Although, women live longer than men, they carry an increased life-long risk of being diagnosed with AD even after adjusting for age and lifespan (Barron and Pike, 2012; Vest and Pike, 2013; Alzheimer’s Association, 2016). AD women tend to exhibit a broader spectrum of dementia-related behavioral symptoms and experience greater cognitive deterioration than men in the progression of the disease (Schmidt et al., 2008; Chapman et al., 2011; Mazure and Swendsen, 2016; Nebel et al., 2018). Although men with AD have a shorter survival time (Burns et al., 1991; Todd et al., 2013; Kua et al., 2014; Wattmo et al., 2014a,b), a meta-analysis of neurocognitive data from 15 published studies revealed that women with AD showed a consistent worse mental deterioration than men with the disease, even when at the same stage of the condition (Irvine et al., 2012). Moreover, AD pathology appears more likely to be clinically expressed as dementia in women than in men (Barnes et al., 2005; Lin and Doraiswamy, 2014).

There are multiple biological hypotheses by which female sex may affect AD. As recently reviewed by the Society for Women’s Health Research Interdisciplinary Network on AD, these could be represented by: (1) genetic factors that have a stronger effect in women [i.e., apolipoprotein E (APOE) genotype]; (2) risk factors that differentially affect men and women (i.e., hypertension); or (3) biological events that are uniquely experienced by women (i.e., menopause, pregnancy; Nebel et al., 2018).

Clinical data suggest that neurological consequences of menopause may trigger more severe symptoms and pathology observed in women with AD compared to men (Barnes et al., 2005; Hua et al., 2010; Skup et al., 2011; Hall et al., 2012; Holland et al., 2013; Lin et al., 2015; Ball and Chen, 2016; Filon et al., 2016; Sundermann et al., 2016a,b, 2017; Jack et al., 2017; Koran et al., 2017; Fisher et al., 2018; Buckley et al., 2019; reviewed in Li and Singh, 2014; McCarrey and Resnick, 2015; Georgakis et al., 2016; Hampel et al., 2018). In support of this, early surgical menopause was associated in women with a 2-fold increase in dementia risk, faster rate of cognitive decline, and more AD pathology (Bove et al., 2014; Fisher et al., 2018). Randomized clinical trials and observational studies found menopause, and particularly the peri-menopause phase, associated with decline in memory function, and increase risk of mild cognitive impairment and dementia (Joffe et al., 2006; Morrison et al., 2006; Greendale et al., 2009; Epperson et al., 2013). Findings from two longitudinal studies reported that peri-menopause was associated with decreased cognitive performance compared to not only pre-menopause, but also post-menopause (Greendale et al., 2009; Epperson et al., 2013), suggesting that the memory impairment was temporary during menopause transition. Recent cross-sectional studies, however, found impairment in verbal memory also in post-menopausal women (Jacobs et al., 2016, 2017; Rentz et al., 2017).

AD pathological hallmarks—Aβ plaques, NFTs, chronic gliosis, and neurodegeneration (Hampel et al., 2018)—are laid down during a prodromal period beginning ~20 years before clinical symptoms appear, which coincides with the time of peri-menopause in women (Jack et al., 2013; Dubois et al., 2016).

Clinical findings suggest that neurological changes during peri-menopause increase the female brain vulnerability to AD. Accordingly, two recent amyloid-PET and magnetic resonance imaging (MRI) studies on cognitively normal women reported increased indicators of AD phenotype—Aβ deposition in frontal and temporal cortex, hypometabolism, and reduced brain volume in AD-vulnerable regions—starting at peri-menopause (Mosconi et al., 2017b) compared to pre-menopause and male sex (Mosconi et al., 2017a,b). This suggests that irregular estrogen fluctuations and neuroendocrine changes unique to peri-menopause, rather than the loss of estrogen at post-menopause, may be the triggering events that increase the susceptibility to AD risk accelerating AD neuropathology and cognitive decline in women.

Observational data link the use of HRT with lower AD risk in women, but clinical trials employing HRT have produced many inconsistent results, largely due to certain caveats such as hormone formulation, timing of therapy, and dose or route of hormone administration as well as studies limited to post-menopausal women (McCarrey and Resnick, 2015; Georgakis et al., 2016).

Ovaries produce and secrete several types of estrogens, including estrone (E1), 17α- and 17β-estradiol (E2), and estriol (E3). 17β-estradiol is the most abundant and potent female gonadal hormone based on binding activity to ERs (Folmar et al., 2002; Blaustein, 2008; Koebele and Bimonte-Nelson, 2015). Most human studies, but not all, have used the HRT formulation consisting in Conjugated Equine Estrogens (CEE; Hersh et al., 2004), which upon absorption and metabolism are primarily converted into 17β-estradiol and equilin (Sitruk-Ware, 2002; Bhavnani, 2003).

The initial results from controlled clinical trials, including the Women’s Health Initiative (WHI), WHI Memory Study (WHIMS), and WHI Study of Cognitive Aging (WHISCA) found that HRT in women may lead to no, or even adverse, effects on cognition and AD risk (Shumaker et al., 2003, 2004; Maki and Henderson, 2012, 2016; Gurney et al., 2014; Hampel et al., 2018). Administration of CEE and progestin medroxy-progesterone acetate after a prolonged period of hypogonadism or menopause diminishes the neuroprotective effect of HRT and enhances neuroinflammation (Shumaker et al., 2003, 2004). Data from the recent WHI publication on the effect of HRT on mortality reported lower risk of dying from AD and dementia for women receiving estrogen, but not estrogen in combination with progestin (Manson et al., 2017). To date, there is no evidence to support that HRT initiated during early post-menopause confer cognitive benefit, but it appears to be safe for cognitive function (Espeland et al., 2013; Gleason et al., 2015; Georgakis et al., 2016; Henderson et al., 2016). However, recent studies have shown that HRT initiated during peri-menopause lowers the risk of AD and has cognitive benefits, especially to hippocampal-mediated memory processes (Henderson et al., 2005; Whitmer et al., 2011; Shao et al., 2012). In the Cache County Memory Study, peri-menopausal women who used HRT had a reduced risk of AD later in life (Zandi et al., 2002; Shao et al., 2012). Data from the WHIMS-Young study and the Kronos Early Estrogen Prevention Study (KEEPS), which only enrolled early menopausal women, support the beneficial effect of HRT on cognitive function early in the menopause transition process (McCarrey and Resnick, 2015). Therefore, the “Window of opportunity” hypothesis was formulated that the beneficial effect of estrogen on cognition and AD depends on women’s age and stage of menopause, and HRT started during post-menopause when a new hormone-receptor equilibrium is already achieved may disturb the established balance (Resnick and Henderson, 2002; Alzheimer’s Association, 2016; Pines, 2016; Pike, 2017; Fisher et al., 2018). Similarly, the “Healthy Cell Bias of estrogen action” hypothesis has been proposed (Brinton, 2008; Gillies and McArthur, 2010). The healthy cell bias postulates that, as cognitive health declines over women’s lifetime, so are the benefits of HRT treatment on the brain. Specifically, the effects of HRT on cognitive function may progress from beneficial to neutral or deleterious over time. In this scenario, estrogen is neuroprotective if neurons are healthy at time of HRT administration. In contrast, estrogen exposure negatively affects neurological functions if neuronal health is compromised. This may explain the initial results from the different WHI studies where HRT administered later in life (and after menopause) had neutral or adverse effects on cognition. Since it is more likely that neurons during peri-menopause are healthier than at post-menopause, the window of opportunity and healthy cell bias hypotheses are inter-related. Yet, the critical cellular and molecular mechanisms underlying the influence of peri-menopause are still awaiting a clear understanding.

## Molecular Mechanisms for the Effect of Peri-Menopause on AD Brain

Estrogens exert their physiological and pathological actions mainly through nuclear estrogen receptors (nERs—ERα, ERβ), and membrane estrogen receptors (mERs) which include ERα, ERβ, G-protein coupled receptor 30 (GPER1 aka GPER30), ER-X, Gq-coupled membrane receptor (Gq-mER; Milner et al., 2001, 2005; McEwen et al., 2012; Hara et al., 2015; Korol and Pisani, 2015; McEwen and Milner, 2017). Each brain region possesses a specific ERs gene expression profile, and activity of each ER is differentially regulated by estrogen, which may account for the sex brain dimorphism (Waters et al., 2009; Mitterling et al., 2010; Li and Singh, 2014). Acting *via* genomic and non-genomic signaling pathways, estrogen has long been known to regulate brain function in both males and females *via* multiple mechanisms of action (Cui et al., 2013). For a review of estrogen actions on the brain, see Spencer et al. (2008); Gillies and McArthur (2010); Luine (2014); Frick (2015); Koebele and Bimonte-Nelson (2017) and McEwen and Milner (2017). In the interest of space, this review article will only discuss biological mechanisms that are related to menopause and AD. For a review of studies on sex differences in mouse models of AD, see Gillies and McArthur (2010); Dubal et al. (2012) and Li et al. (2014).

Changes in estrogen levels during menopause have been associated with mitochondrial dysfunction, synaptic decay, and neuroinflammation. Localization of ERs directly within mitochondria suggests that estrogen may regulate **mitochondrial activity** *via* genomic mechanism, but also affect its function directly by modulating mitochondrial respiration and mitochondrial DNA transcription (Klinge, 2017). This is particularly relevant in the brain as mitochondrial dysfunction plays a role in aging and neurodegenerative diseases, specifically in AD (Barja, 2004; Cantuti-Castelvetri et al., 2005; Kujoth et al., 2007). In the 3xTgAD mouse model of AD, Dr. Brinton lab showed that there is a significant correlation between decreased mitochondrial and glucose metabolism and Aβ load in the hippocampus of aged or ovariectomized OVX females with respect to intact females (Brinton, 2008; Yao et al., 2010, 2012; Ding et al., 2013a,b). However, the hypometabolism in the hippocampus of aged and OVX females was observed across all ages, and in both 3xTgAD and non-transgenic mice suggesting that these mechanisms may influence AD, but are not exclusive to it (Yao et al., 2012; Ding et al., 2013a). Accordingly, pre-clinical and human brain imaging studies showed that Aβ and tau pathology precede metabolic changes and cognitive deficits in AD (Landau et al., 2012; Jack et al., 2013), supporting the idea that mechanisms other than hypometabolism may act during menopause to accelerate the initiation of AD.

Another possible mechanism for menopause effect on AD may be through the estrogen modulation of **neuronal excitability and synaptic plasticity** (Spencer et al., 2008; Spencer-Segal et al., 2012; Baudry et al., 2013; Arevalo et al., 2015; Hara et al., 2015; Waters et al., 2015), which are major mechanisms underlying learning and mnemonic processes. Indeed, high levels of 17β-estradiol during the proestrus phase of the estrous cycle increases hippocampal excitability, long term potentiation (LTP), and remodels dendritic spines in female rodents (Woolley and McEwen, 1992; McEwen et al., 2001; Spencer et al., 2008; Mukai et al., 2010; Broestl et al., 2018). This suggests that irregular estrogen fluctuations during peri-menopause and the following decline in estrogen levels may profoundly impact neuronal activity in females. The effects of menopause transition on neuronal activity may also be related to the estrogen action on neurotrophins, including brain-derived neurotrophic factor (BDNF; Spencer et al., 2008, 2010; Spencer-Segal et al., 2011; Wei et al., 2017), which is an important regulator of synaptic plasticity in the brain (Fisher et al., 2018). BDNF is neuroprotective against Aβ-induced cell death, and its levels are reduced in hippocampus and serum of individuals with AD (Laske et al., 2006a,b; Arancibia et al., 2008; Tapia-Arancibia et al., 2008; Nagahara et al., 2009; Nagahara and Tuszynski, 2011). Interestingly, recent studies on carriers of the BDNF single-nucleotide polymorphism Val66Met showed an increase in AD risk in women but not in men, and that women respond differently to estrogen in hippocampal-related working memory tasks compared to non-carrier controls (Wei et al., 2017, 2018; Fisher et al., 2018). Serum levels of BDNF significantly decrease in menopause women and during aging. In rodent models, aging and OVX induce a significant reduction in hippocampal BDNF expression which is ameliorated by HRT.

Other than affecting neurophysiology in higher cognitive brain regions—hippocampus, frontal cortex, basal forebrain, and striatum—estrogen also influences the neuropathology of AD including seeding of parenchymal Aβ plaques, intracellular NFTs, and chronic inflammation (Brinton, 2008; Hirata-Fukae et al., 2008; Lee et al., 2014; Au et al., 2016; McEwen and Milner, 2017; Merlo et al., 2017; Fisher et al., 2018).

Amyloid precursor protein (APP) is processed by two competing pathways, the non-amyloidogenic pathway *via* α-secretase, and the amyloidogenic *via* β-secretase (BACE1), which produces the **toxic β-APPs and Aβ40/Aβ42 peptides** (Baranello et al., 2015). Estrogen can reduce Aβ deposits by favoring the non-amyloidogenic pathway and reducing BACE1 levels, promoting Aβ glial phagocytosis, and regulating the major enzymes involved in Aβ degradation (Jaffe et al., 1994; Xu et al., 1998, 2006; Li et al., 2000; Manthey et al., 2001; Levin-Allerhand et al., 2002; Joffe et al., 2006; Yao et al., 2007; Liang et al., 2010; Zhao et al., 2011; Lee et al., 2014). In hippocampus and prefrontal cortex (PFC) of AD patients, estrogen loss exacerbates deposition of Aβ and NFTs, which induces synaptic dysfunction (Pike, 2017). Following OVX, levels of Aβ peptide and Aβ plaque burden are increased in different transgenic mouse models of AD in females compared to males, and are rescued by estradiol treatment (Callahan et al., 2001; Levin-Allerhand et al., 2002; Zheng et al., 2002; Carroll et al., 2007, 2010). In the 3xTgAD mouse model there is a significant correlation between decreased mitochondrial and glucose metabolism and Aβ load in the hippocampus of aged or OVX females with respect to intact females (Yao et al., 2010, 2012; Ding et al., 2013a; Ding et al., 2013b).

Deposition of NTFs **containing hyperphosphorylated tau protein** may also be an early event during the prodromal phase of AD, which correlates significantly with cognitive symptoms later in life (Nelson et al., 2009; Jack et al., 2013; Wang and Mandelkow, 2016). In the presence of brain tau pathology, women have an increased risk of AD compared to men (Barnes et al., 2005). In mouse models overexpressing mutant tau, females show higher levels of tau pathology and more severe cognitive deficits compared to males (Asuni et al., 2007; Yue et al., 2011; Buccarello et al., 2017), although the animal’s ages spanned from young to old (5–15 months). This discrepancy may be explained by the mouse models used or by the concurrence of estrogen-independent mechanisms. Most pre-clinical studies agree that estradiol reduces levels of hyperphosphorylated tau *via* ERs, though opposing roles for ERα and ERβ were reported (Merlo et al., 2017).

Several lines of evidence support the pivotal role of **chronic inflammation** in the initiation and progression of AD (Frautschy et al., 1998; Benzing et al., 1999; Parachikova et al., 2007; Kraft et al., 2013; Christensen and Pike, 2015; Heneka et al., 2015; Matarin et al., 2015; Au et al., 2016; Song et al., 2016; Villa et al., 2016; Wang et al., 2016; Czirr et al., 2017; Jevtic et al., 2017; Liu et al., 2017). An increase in density of resting microglia precedes Aβ plaque formation, and increased activated microglia is observed around Aβ plaques in the brain of transgenic models of AD (Heneka et al., 2015; Wang et al., 2015; Manocha et al., 2016; Jevtic et al., 2017). Inflammation is another risk factor for AD that varies by sex (Hanamsagar and Bilbo, 2016). The expression of genes related to inflammation increases with age more in the forebrain of menopausal women than men and pre-menopausal women (Sárvári et al., 2012; Christensen and Pike, 2015). Acting *via* ERα and ERβ on glial cells, estrogen regulates glial response to neurotoxins and promotes Aβ removal (Li et al., 2000; Blurton-Jones and Tuszynski, 2001; Au et al., 2016; Villa et al., 2016; Merlo et al., 2017). At menopause, estrogen level fluctuations affect conversion of microglia phenotype from resting state to reactive state (Sárvári et al., 2012). In rodents, aging or OVX induce changes in ERα and ERβ levels on astrocytes which leads to impaired neurotrophic responses to estrogen (Rozovsky et al., 2002; McAsey et al., 2006; Morgan and Finch, 2015). Accordingly, reactive astrogliosis and microglia reactivity marker genes are up-regulated in OVX female mice (Sárvári et al., 2012).

Furthermore, besides directly influencing brain structures regulating cognitive function, hormonal and neurological changes during menopause transition may indirectly influence other risk factors that differentially affect men and women, such as APOE genotype and hypertension (see also paragraph on the use of VCD mouse model to study hypertension; Nebel et al., 2018). For instance, the most well-established genetic risk factors for late onset AD is the presence of a common allele ε4 in the **APOE gene** (Corder et al., 1993; Coon et al., 2007), which encodes for a lipid-binding protein crucial in triglycerides and cholesterol transport to neurons (Puglielli et al., 2003; Bu, 2009; Leduc et al., 2010). APOE4 genotype increases risk of developing AD over the other alleles APOE2 and APOE3 by 5–10 years (Noguchi et al., 1993; Ossenkoppele et al., 2015; Fisher et al., 2018), and was reported to increase Aβ deposition and oligomer formation, as well as phosphorylated tau within neurons (Riedel et al., 2016; Fisher et al., 2018). The APOE4 allele shows sex-dependent effects whereby it is a stronger risk factor for AD in female than male carriers of same age (Farrer et al., 1997; Mortensen and Hogh, 2001; Beydoun et al., 2012; Altmann et al., 2014; Neu et al., 2017). APOE4 women also show a sharper decline in cognitive function and have a greater Aβ brain pathology than APOE4 men (Mortensen and Hogh, 2001; Corder et al., 2004; Beydoun et al., 2012; Fisher et al., 2018). *In vitro* and *in vivo*, activation of ERα up-regulates levels of ApoE mRNA and protein, whereas selective ER agonists down-regulate ApoE mRNA and protein levels in rat hippocampal neurons (Wang et al., 2006). Altogether, this may suggest that HRT could constitute a possible approach to provide therapeutic benefits and/or reduce the risk of developing AD in APOE4 carriers. However, studies on the use of estrogen formulations have provided inconclusive results thus far highlighting a complex interaction between estrogen, APOE and AD (Depypere et al., 2016; Riedel et al., 2016).

In conclusion, there is enough evidence to support that widely fluctuating changes in estrogen-ER response network may underlie the increased susceptibility of female brains to AD conferred by menopause transition. In many women, the brain compensates for these changes during peri-menopause. However, it is likely that in the presence of other AD risk factors for some women this adaptive compensation is diminished, which gives rise to the increased the susceptibility to AD pathogenesis.

## Rodent Models of Menopause

Despite continuous advances in understanding AD pathophysiology, it has been challenging to systematically evaluate the biological mechanisms underlying the influence of menopause on AD in the human population, and clinical research continues to have many inconsistent results and unresolved issues. Pre-clinical rodent models of menopause have proven useful to start teasing out the neurological changes that during menopause may increase the female brain vulnerability to AD. The disparity between animal study results and epidemiological and clinical data has highlighted the need to address the strengths and weaknesses of current animal models of menopause and their use to predict therapeutic outcomes.

**Figure 1:**
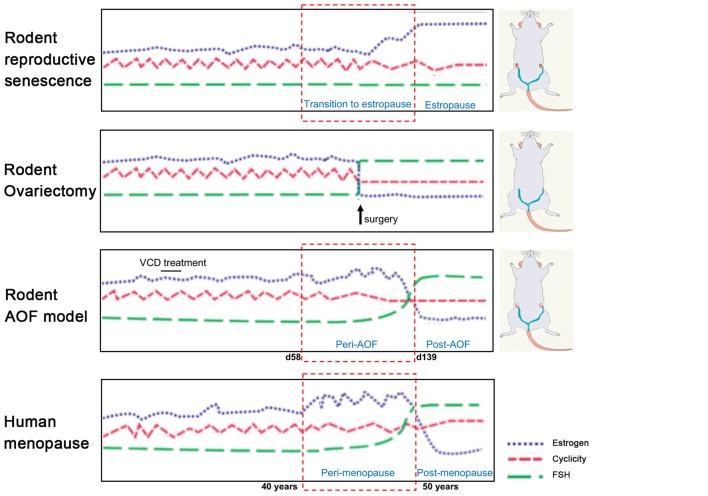
Rodent models of menopause. (On the left) Schematic illustration of hormonal levels and cyclicity in the different rodent models of menopause in comparison with the human menopause process. (On the right) Schematic drawings of the models. In the senescence model, ovaries are preserved, and animals have regular estrous cycles in adulthood before transitioning to estropause in middle-age. In the ovariectomy (OVX) model, ovaries are surgically removed and there is complete and abrupt loss of ovarian hormones. In the innovative vinylcyclohexene diepoxide (VCD) model, ovaries are preserved, and ovarian follicles are depleted inducing a progressive transition to ovarian failure with gradual hormonal changes similarly to the human menopause process. Image was adapted with permission from Jackson lab (www.jax.org).

The most employed models, ovary-intact aging (reproductive senescence) and ovariectomy present several drawbacks that limit our understanding of the neurological underpinning of menopause transition. These models will be discussed in the following paragraphs in view of their application in AD research and compared to the more recently developed model of AOF (Figure 1).

### Reproductive Senescence

The ovary is the main site of female sex hormone production. The mammalian ovary has two major functions: (1) differentiation, maturation, and release of oocytes for fertilization (McGee and Hsueh, 2000); and (2) production and sequential secretion of the hormones estrogen, progesterone, and inhibin A and B that regulate the hypothalamus–pituitary–gonadal axis in a repetitive process of follicle development, ovulation, corpus luteum formation and regression during the menstrual/estrous cycle (Peters et al., 1975; Hirshfield, 1991). At birth, the mammalian ovary carries a finite number of immature oocyte-containing follicles, which represent a limited pool of germ cells for reproduction. Following follicular maturation, oocytes are released during ovulation to be fertilized. Most follicles do not reach maturity and go through cell death named atresia. Physiologically, menopause occurs following depletion of all follicles in the ovary (McGee and Hsueh, 2000).

Women have a menstrual cycle, whereas female rodents have an estrous cycle which occurs every 4–5 days and consists of proestrus, estrous, metestrus, diestrus phases that involve similar hormonal fluctuations seen in the human cycle (Goldman et al., 2007). Similar to women, rodents experience natural hormonal fluctuations that emerge at middle age (9–12 months of age) which involves irregular cycles with prolonged diestrus phases and possible absence of ovulation (Maffucci and Gore, 2006; Downs and Wise, 2009; Finch, 2014). Around 1 year of age, differently from the human menopause, rodents transition into a state of extended estrous phases (estropause) with cessation of reproductive cycles. Eventually, at about 16–18 months of age, rodents transition into an acyclic, anestrous state involving persistent estrous and complete halt of ovulation (Chakraborty and Gore, 2004; Maffucci and Gore, 2006). Stages of pseudopregnancy with irregular ovulation and dysregulation of estrogen levels are also common during the rodent estropause. Interestingly, the ovary also produces testosterone. However, levels of ovarian testosterone are not impacted by menopause transition, unlike estrogens and progestins, but declines slowly with age (Burger, 2002; Davis and Wahlin-Jacobsen, 2015). Research conducted over the past 20 years in the ovary-intact aging model supports the notion that female brain vulnerability to AD is associated with loss of ovarian hormones after menopause. For instance, several groups reported an increase in Aβ deposition in the brains of 1-year-old, and older, females compared to age-matched males and young females in rodent models of AD (Zheng et al., 2002; Wang et al., 2003). Using the aging model, the Brinton group has provided gene expression and proteomic evidence of an early shift in mitochondrial and lipid metabolism in the hippocampus of females tested staring at ~9 months of age compared to age-matched males and young females in the 3xTgAD mouse model (Yao et al., 2009; Ding et al., 2013a; Zhao et al., 2016). However, this does not rule out the possibility of an influence of the aging process itself and high estrogen levels in the elderly rodent systems used.

In conclusion, the rodent reproductive senescence model is characterized by multiple features found in the human menopause including: (1) retention of ovarian tissue; (2) irregular cycling and steroid hormone fluctuations; and (3) irregular fertility. However, because rodents do not exhibit significant depletion in the pool of ovarian follicles at the onset of reproductive senescence, circulating ovarian hormone levels do not present a drastic reduction (Dubal et al., 2012). This rodent model of aging contrasts from the human menopause, where levels of estrogens are very low or undetectable (Chakraborty and Gore, 2004).

### Ovariectomy Model

Ovariectomy, or bilateral surgical removal of the ovaries, is a well-established model of menopause (Olson and Bruce, 1986; Maffucci and Gore, 2006). In this model, rodents can be OVXed at different ages that correlate with different life stages. For example, females may be OVXed at 2–6 months of age with regular estrous cycles, 10 months at the beginning of the acyclicity, and at 18 months of age at the beginning of persistent estrous. This is an ideal model to study the effect of ovarian hormones deficit and influence of exogenous hormonal treatments on the brain. Experimental interventions may occur either at the time of OVX or commence once 17β-estradiol has reached a low to non-detectable level in the plasma, which typically occurs within 1–2 weeks (Marques-Lopes et al., 2018).

Using this model, many laboratories have studied the effect of estrogen loss, and 17β-estradiol and other hormonal formulations, on cognitive function in normal wild type and AD rodents (Diaz Brinton, 2012; Li et al., 2014; Koebele and Bimonte-Nelson, 2016). For instance, administration of 17β-estradiol to OVX females have shown to increase performance in spatial memory behavioral tasks (Koebele and Bimonte-Nelson, 2016; Koebele et al., 2017). As mentioned above, in the 3xTgAD mouse model of AD, there is a significant correlation between decreased mitochondrial and glucose metabolism and Aβ load in the hippocampus of OVX females with respect to intact females (Yao et al., 2009, 2012; Ding et al., 2013a,b). The shift in energy metabolism was associated with an increase in Aβ deposition in OVX females compared to males and ameliorated by treatment with 17β-estradiol (Callahan et al., 2001; Levin-Allerhand and Smith, 2002; Zheng et al., 2002; Yue et al., 2005; Yao et al., 2012; Ding et al., 2013b; Lee et al., 2014).

In the OVX model, some shortcomings may influence the interpretation of data: (1) age of mice at OVX; (2) dose, duration, and mode of administration of hormonal treatment; and (3) differential animal hormonal responsiveness depending of time elapsed since OVX (Gibbs, 1997; Rapp et al., 2003; Maffucci and Gore, 2006; Scharfman et al., 2007). For instance, several studies have addressed these issues and support that estrogen sensitivity decreases when greater time has lapsed since OVX or with increasing age making it difficult to compare across experiments (Morrison et al., 2006; Smith et al., 2010; Koebele et al., 2017).

While the OVX model provides insights into the role of estrogen and other gonadal hormones separately from aging as a confounding factor, this has two drawbacks. First, in addition to estrogens, OVX depletes other hormones that have important roles in menopause and can impact brain function (Maffucci and Gore, 2006; Rocca et al., 2011). Second, since the majority of menopausal women have intact ovaries, the abrupt loss of gonadal hormones models surgical menopause, but not natural transitional menopause. This does not allow to shed light on the progressive changes in estrogen responsiveness that occur when the natural cycle of circulating estrogens are disrupted at peri-menopause (Nejat and Chervenak, 2010; Shuster et al., 2010; Van Kempen et al., 2011). In support of this, Koebele and Bimonte-Nelson (2017) have shown that in rats, the model of ovarian hormone loss influences spatial memory performance and response to estrogen therapy, supporting the importance of studying surgical and transitional menopause independently (Acosta et al., 2009, 2010).

### The Accelerated Ovarian Failure (AOF) Model

Until recently, aging and OVX have been the primary rodent models of menopause to examine effects of ovarian hormone loss on cognition and AD in females (Koebele and Bimonte-Nelson, 2016). Although these models have furthered our understanding of estrogen actions on the central nervous system, as reported above, the rodent aging and OVX do not fully recapitulate the transitional events of human menopause (Van Kempen et al., 2011). The lack of an adequate model that includes a progressive transition through menopause has hindered studies on how irregular hormonal changes during peri-menopause impact the female brain susceptibility to AD, and the identification of the molecular mechanisms underlying this phenomenon that can be targeted to prevent or delay the disease. Lack of an optimal model that could also dissociate the effect of menopause from aging has limited the studies on the timing of hormonal replacement therapies and how these can influence disease outcomes.

**Figure 2:**
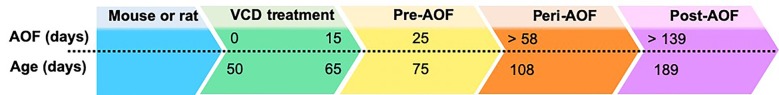
Accelerated ovarian failure (AOF) model timeline. AOF stages can be calculated in days following initiation of VCD treatment (15 days total). Days above the dotted line indicate time after start of VCD treatment, while days below the line indicate the animal chronological age. Pre-, peri, and post-AOF stages are indicated by the arrowhead boxes.

In early 2000s, the Hoyer group developed the innovative model of AOF that successfully replicates the human peri- and post-menopause stages, including irregular estrous fluctuations at peri-AOF (Mayer et al., 2004, 2005; Williams, 2005; Van Kempen et al., 2011, 2014), and acyclicity at post-AOF stage with very low estrogen levels, and follicle-stimulating hormone (FSH) and luteinizing hormone (LH) levels raising accordingly (Mayer et al., 2002; Van Kempen et al., 2011; Brooks et al., 2016; Marques-Lopes et al., 2018). In this model, 15-day administration of low doses of the 4-VCD (130–160 mg/Kg in 0.5% dimethyl sulfoxide in sesame oil; i.p.) induces selective depletion of ovarian primary and primordial follicles (Lohff et al., 2006; Acosta et al., 2009; Van Kempen et al., 2011, 2014; Brooks et al., 2016; Koebele and Bimonte-Nelson, 2016). For a mechanistic review, see Hoyer and Sipes (1996, 2007) and Hoyer et al. (2001). Following VCD treatment, mature follicles deplete with normal estrous cycles (Flaws et al., 1994; Mayer et al., 2002; Lohff et al., 2006) resulting in a progressive transition to acyclicity and ovarian failure (Mayer et al., 2004). In addition to reducing confounds associated with surgical manipulations, the AOF model maintains the presence of ovarian tissue and presents a peri-AOF stage which importantly parallels human peri-menopause (Mayer et al., 2004; Rivera et al., 2009). Another advantage of this model is that it allows for the longitudinal study of the effects of hormone levels dissociated from the effects of aging in young animals (Danilovich and Ram Sairam, 2006; Koebele and Bimonte-Nelson, 2016). The drawback of this model is that VCD is toxic at higher doses (National Toxicology Program, 1986). However, in a laboratory setting, administration of low doses of VCD does not negatively affect peripheral tissues, including liver and kidney function and organ weights (Devine et al., 2001; Hoyer et al., 2001; Mayer et al., 2005; Haas et al., 2007; Sahambi et al., 2008; Wright et al., 2008; Frye et al., 2012; Van Kempen et al., 2014), and does not appear to cross the blood–brain barrier (Van Kempen et al., 2011, 2014). Work from the Milner group established that this model is suitable for longitudinal studies (Van Kempen et al., 2014), and does not have any direct effects on induction of inflammation markers in brain areas inside (e.g., hippocampus and hypothalamus) and outside (e.g., circumventricular organs) the blood–brain barrier (Van Kempen et al., 2014). Based on the assessment of ovarian follicle depletion and responses of plasticity markers in brain areas known to be estrogen responsive, timepoints for pre-AOF (~25 days after first injection), peri-AOF (>58 days post-injection), and post-AOF (>139 days post-injection) stages have been established corresponding to the human pre-, peri-, and post-menopause, respectively (Mayer et al., 2004; Lohff et al., 2006; Van Kempen et al., 2011, 2014; Marques-Lopes et al., 2018; Figure 2). Length of peri-AOF period and beginning of post-AOF can be manipulated by varying the duration of VCD treatment (Brooks et al., 2016).

Several laboratories have implemented this model to study cognition, cardiovascular disease, insulin resistance, atherosclerosis, bone loss, anxiety (Van Kempen et al., 2014), and hypertension (see next paragraph). A fairly recent summary of VCD doses and organs evaluated in studies applying the AOF model can be found in Van Kempen et al. (2011) and Marques-Lopes et al. (2018). A single study using the AOF has been reported in Tg2576 mouse model of AD. Golub et al. (2008) treated female mice with VCD at 60–75 days of age to induce AOF, and found no significant improvement of estrogen replacement in cognitive performance or Aβ plaque load in the hippocampus and anterior cortex of post-AOF females. The study was affected by low power due to small sample size and measured the effect of estrogen only on late post-AOF mice at 15 months of age, thus missing the opportunity to quantify the influence of peri-AOF on AD phenotype and introducing aging as a variable during data collection. Therefore, the influence of peri-AOF on neuropathology and phenotype in AD model has not been tested and cannot be ruled out. Nevertheless, the AOF models has been used to study cognitive changes in both mice and rats, which suggested a need for caution in extrapolating data on cognitive function from OVX models to a VCD model, and demonstrated the importance of studying transitional and surgical menopause independently (Acosta et al., 2009, 2010; Koebele and Bimonte-Nelson, 2017; Koebele et al., 2017).

In summary, studies employing VCD-treated animals highlight that this model of AOF is extremely useful for modeling biological mechanisms and disease states associated with peri- and post-menopause, and that studies employing the VCD model will be particularly valuable to validate the most promising findings generated in the aged and OVX models of AD.

### Lessons From Hypertension Studies as an Illustration of Rodent AOF Model Utilization

Although works employing the AOF in AD animals are lacking, this model has been used to study sex dimorphism in other diseases and in risk factors of AD, such as the study of the influence of menopause transition on chronic elevated blood pressure (hypertension; Van Kempen et al., 2011; Iadecola, 2014; Wiesmann et al., 2017; Marques-Lopes et al., 2018). Given that some AD risk factors, like hypertension, are modifiable, determining the extent to which sex differences contribute to differential vulnerability may present opportunities for the development of more targeted and efficacious therapies. A large body of literature supports the deleterious role of hypertension in midlife as a risk factor for AD in both men and women (Feldstein, 2012; Davey, 2014a,b; Iadecola, 2014; Iadecola et al., 2016; Kehoe, 2018). There is both clinical and pre-clinical evidence that hypertension induces cerebrovascular damage that causes the reduction of Aβ removal from the brain, and activates chronic inflammation, factors that accelerate the neuropathology and cognitive impairment in AD (Zlokovic, 2011; Carnevale et al., 2012; Shah et al., 2012; Attems and Jellinger, 2014; Kruyer et al., 2015; Faraco et al., 2016).

Sex differences in blood pressure control have been extensively reported (Lima et al., 2012; Sandberg and Ji, 2012). Men with hypertension seem to have a higher risk of developing AD than pre-menopausal women, but this relationship is inverted during the menopause transition and postmenopausal women have a higher risk of AD than age-matched men (Burt et al., 1995a,b; Zanchetti et al., 2005; Yanes and Reckelhoff, 2011; Lima et al., 2012). Notably, estrogen influence on blood pressure involves regulation of a number of brain regions including the ventrolateral medulla, nucleus of solitary tract, and paraventricular nucleus of the hypothalamus (McEwen et al., 2012). Within this circuitry, there are many potential sites by which estrogen can interact with renin-angiotensin system and molecular signaling pathways critical for regulation of brain cardiovascular circuits (McEwen et al., 2012; Maranon et al., 2014; McEwen and Milner, 2017).

It is now established that young female mice are protected from slow pressor Angiotensin II (AngII)-induced hypertension compared to age-matched males (Girouard et al., 2009; Xue et al., 2009, 2013, 2014; Marques-Lopes et al., 2014, 2015, 2017; Van Kempen et al., 2015). An increasing number of studies using either the senescence or OVX models have shown that in “post-menopause-like” state females are more susceptible to AngII-induced hypertension to a magnitude similar to that observed in males (Tiwari et al., 2009; Capone et al., 2012; Sandberg and Ji, 2012; Coleman et al., 2013; Marques-Lopes et al., 2014, 2015). These studies provide a first indication that estrogen may play a critical role in protection against hypertension. Interestingly, by using the AOF model, work from Dr. Milner group showed for the first time that peri-AOF is the critical time for the susceptibility to AngII-induced hypertension (Marques-Lopes et al., 2014, 2017; Van Kempen et al., 2016). While the data obtained employing the aging and OVX models only suggested that estrogen plays a role in hypertension in rodents, in the studies by the Milner group, the use of the AOF model uniquely allowed the testing of the hypothesis that irregular estrogen fluctuations during AOF transition, rather than loss of estrogen at post-AOF, may be responsible for the observed increase in susceptibility to AngII-induced hypertension. Furthermore, it was shown that estradiol activation of ERβ in the paraventricular nucleus of the hypothalamus (PVN) attenuates the glutamate-induced increase in blood pressure (Gingerich and Krukoff, 2006). To better understand the link between estrogen and hypertension, Milner et al. studied the changes in subcellular localization of the glutamate N-Methyl-D-aspartate (NMDA) receptor subunit GluN1 in ERβ-positive neurons of the PVN following slow pressor AngII treatment in males and females using both the senescence and AOF models (Marques-Lopes et al., 2014, 2017). In both models, AngII-treated young females, males, and peri-AOF females show decreased total density of GluN1 in ERβ dendrites of PVN, whereas aged females and post-AOF females had an increase in total density. However, in males and peri-AOF females, plasmalemmal affiliation of GluN1 was increased, while it was unchanged in post-AOF females. As for the studies by Acosta et al. (2009, 2010) on the influence of the menopause model used in cognitive tasks, these findings indicate that distinct neurobiological processes underlie AngII-induced hypertension in aging and AOF, which may arise from the differences in estrogen levels between aged and AOF female mice.

## Conclusion

The multi-factorial nature of AD neuropathology and symptomatology has taught us that a single therapeutic approach will most likely not fit all. Studying the effects of sex differences and menopause will lead to the development of novel targeted precision medicine approaches that take sex and hormonal status into account.

Although substantial advances in medicine and research for AD have been made over the past years, the degree to which human studies can understand the neurological mechanisms of menopause in AD is limited. Rodent models of menopause have proven very useful to start dissecting the key molecular mechanisms underlying the influence of menopause transition on AD. In particular, the innovative model of AOF can provide a valuable approach to the study of physiological changes that more closely parallel to the ones of human menopause. The advantages of the AOF as a model of transitional menopause make it an ideal choice for studies of menopause and HRT in mouse AD models.

## Author Contributions

RM performed literature search, prepared and reviewed the manuscript.

## Conflict of Interest Statement

The author declares that the research was conducted in the absence of any commercial or financial relationships that could be construed as a potential conflict of interest.
